# Construction of a High-Density Genetic Map and Quantitative Trait Locus Mapping in the Manila clam *Ruditapes philippinarum*

**DOI:** 10.1038/s41598-017-00246-0

**Published:** 2017-03-22

**Authors:** Hongtao Nie, Xiwu Yan, Zhongming Huo, Liwen Jiang, Peng Chen, Hui Liu, Jianfeng Ding, Feng Yang

**Affiliations:** 1 Engineering and Technology Research Center of Shellfish Breeding in Liaoning Province, Dalian Ocean University, Dalian, 116023 China; 2 Key Laboratory of Mariculture and Stock Enhancement in North China’s Sea, Ministry of Agriculture, Dalian Ocean University, Dalian, 116023 China

## Abstract

Genetic linkage maps are indispensable tools in a wide range of genetic and genomic research. With the advancement of genotyping-by-sequencing (GBS) methods, the construction of a high-density linkage maps has become achievable in marine organisms lacking sufficient genomic resources, such as mollusks. In this study, high-density linkage map was constructed for an ecologically and commercially important clam species, *Ruditapes philippinarum*. For the consensus linkage map, a total of 9658 markers spanning 1926.98 cM were mapped to 18 sex-averaged linkage groups, with an average marker distance of 0.42 cM. Based on the high-density linkage map, ten QTLs for growth-related traits and shell color were detected. The coverage and density of the current map are sufficient for us to effectively detect QTL for segregating traits, and two QTL positions were all coincident with the closest markers. This high-density genetic linkage map reveals basic genomic architecture and will be useful for comparative genomics research, genome assembly and genetic improvement of *R. philippinarum* and other bivalve molluscan species.

## Introduction

The Manila clam *Ruditapes philippinarum* is one of the most exploited marine bivalves worldwide with a production of 3.9 million metric tons in 2013^1^. In China it has been farmed since the 1980s, with the annual yield remaining at around 3.0 million metric tons in recent years. It is also extensively cultivated in northern and southern Asia and Europe (United Nations Food and Agriculture Organization (FAO) Fishery Statistics, 2015). As a burrowing bivalve in intertidal zones, *R. philippinarum* is likely to be affected by marine environmental pollution, especially heavy metal pollution. As a result, *R. philippinarum* is often considered as an indicator organism of marine sediment environmental pollution^2^. Despite its economic and ecological importance, the genetic study of *R. philippinarum* lags far behind that of *Crassostrea gigas*, for which the whole genome has recently been sequenced and characterized^3^. Comparatively, the genetic exploration of *R. philippinarum* is very limited, with the transcriptome of only a few RNAs being sequenced so far^4–8^. A genetic linkage map is a critical and indispensable tool for genetic and genomic research^9^, which can provide a foundation for identification of genomic loci linked to phenotypic variants, mapping of quantitative trait loci (QTL) and even anchoring genomic sequence scaffolds^10,11^. Unfortunately, no genetic linkage map for *R. philippinarum* has yet been produced.

A high-density genetic linkage map is a useful tool for genome assembly, as well as for mapping QTL of economically important traits^10^. The low efficiency and high cost of traditional strategies for marker development have become the major obstacles restricting the usage of these techniques in the generation of high-density genetic linkage maps^12^. Recent advances in next-generation sequencing (NGS) technologies have enabled the identification of sufficient genetic molecular markers at a reasonable price, thereby promoting the development of several high-throughput single-nucleotide polymorphisms (SNPs) genotyping methods. Recently, genetic linkage maps have been constructed for many aquaculture species, such as Atlantic salmon^13^, grass carp^14^, catfish^15^, rainbow trout^16^, pacific white shrimp^10^, black tiger shrimp^17^, sea cucumber^11^, scallop^18^, oyster^19^ and others. A genetic map with sufficient density and resolution is one of the essential prerequisites for marker-assisted selection, comparative analysis of genomic synteny, fine mapping of QTL, positioning of candidate genes and facilitating the chromosome assignment of whole-genome scaffolds^11^. Consequently, a high-density linkage map is necessary for genomic and genetic studies in the clam species *R. philippinarum*.

NGS technology has extended our ability to conduct *de novo* genome sequencing and high-density linkage construction for non-model species. One of the major methods used to construct high-density linkage maps, called genotyping by sequencing (GBS) technology, has been widely used in non-model species^20^. This technology enables accurate and high-throughput collection of massive amounts of sequence data^21^, including tens of thousands of SNPs and has been used to identify SNPs segregating between cave-dwelling and surface-dwelling morphs of *Astyanax mexicanus*^20^. GBS utilizes deep Illumina sequencing of restriction enzyme-nicked genomic DNA libraries that are uniquely barcoded for each member of an experimental pedigree. This technique is optimized to avoid inclusion of repetitive portions of the genome and is extremely specific and highly reproducible^22^. Aquatic organisms are well-represented among studies using GBS and other restriction site-associated DNA sequencing (RAD-seq)–based methodologies^23^. The majority of GBS studies in aquatic animals have focused on fish species of commercial^24,25^ or conservational concern^26–28^. In this study, we adapted this technology to construct a high-density linkage map for genomic and genetic studies in the marine bivalve *R. philippinarum*.

We constructed a high-density linkage map for the first time to investigate the genomic and genetic architecture of *R. philippinarum*. Based on the resulting high-density linkage map, QTL mapping was conducted to detect markers related to growth traits.

## Results

### Restriction enzyme selection for library construction

Before construction of the sequencing library, restriction enzymes were evaluated based on the predicted number of tags, length of fragments, and distribution across the reference genome. After scanning the entire genome, a combination of three restriction enzymes, *MseI* (*TTAA*), *NlaIII* (*CATG*), and *EcoRI* (*GAATTC*), was selected for GBS library construction. According to their restriction sites, the predicted sizes of the DNA fragments ranged from 240 to 265 bp and the number of tags was 103,748 with only 2.52% repetitive tags. These tags covered 1,122,973,377 bp (99.69%) of the genome and were distributed across 27,220 scaffolds, accounting for 88.35% of total scaffolds.

### GBS-tag generation and marker genotyping

A total of 73.46 Gb of raw data containing 255,042,108 paired-end reads were generated by sequencing the parents and 119 of their progeny. After data filtering, 94.64% of reads were high quality, with an average Q20 ratio of 97.80% and a GC content of 34.73% (Supplementary Table S1). The parents were sequenced at a higher level to enhance the chances of detecting more SNP markers. Finally, clean data covering 4,181,609,952 (99.98%) and 4,002,681,024 (99.97%) bp were obtained for the female and male parents, respectively. For each individual, the clean data ranged from 264,085,056 to 4,181,609,952 bp, with an average of 607,042,373 bp. The average number of total reads for parents and offspring were 14,208,839 and 1,904,406, respectively (Supplementary Table S2). High-quality clean reads were aligned against the clam genome. Consequently, 14,519,479 paired end clean reads were obtained for the female parent and 13,898,198 paired end clean reads for the male parent. Only reads aligned to unique positions on the reference genome were retained for the subsequent SNP calling and genotyping.

**Figure 1:**
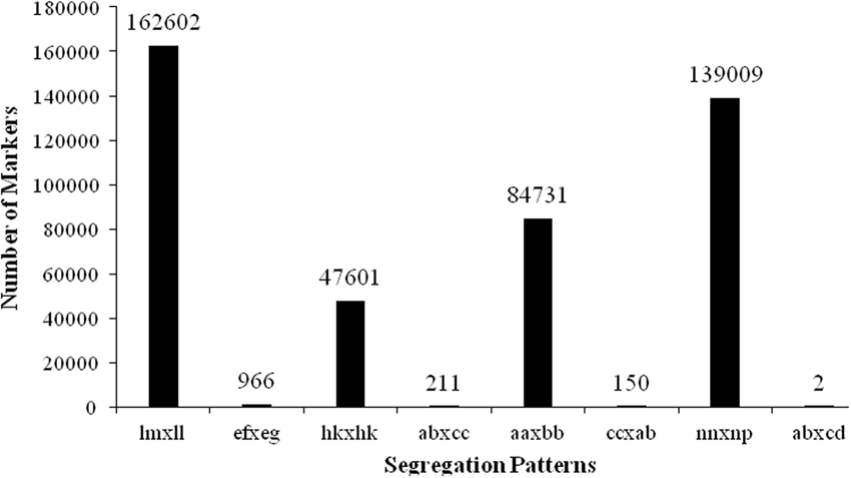
Statistics of genotyped GBS markers in eight segregation patterns.

SNP calling of the two parents and F1 individuals was performed with SAMtools^29^. In total, 1,636,328 and 1,139,671 SNPs were detected in the female and male parents, respectively (Supplementary Table S3). For F1 individuals, an average of 734,852 SNPs was discovered for an individual progeny. These SNPs could be classified into eight segregation types according to the CP model in JoinMap 4.0. As shown in Fig. 1, eight patterns were detected. Among them, four major patterns including lm × ll, nn × np, hk × hk, and aa × bb accounted for nearly 99.7%, while the other three patterns, ab × cc, cc × ab, and ef × eg, only accounted for 0.3%. The type aa × bb was excluded, as it was homozygous in both parents and resulted in no segregation in F1 individuals. Only segregation types lm × ll, nn × np, and hk × hk were selected for genotyping in F1 individuals.

### Genetic linkage map construction

**Figure 2:**
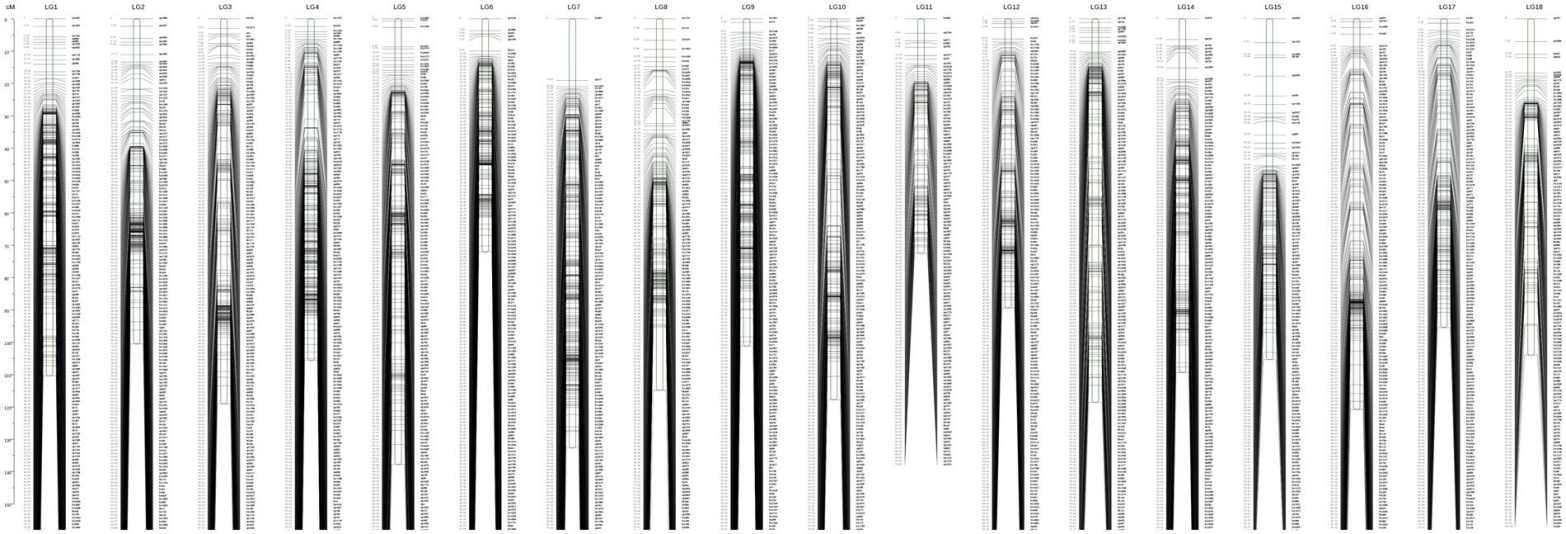
The high-density consensus linkage map of *R. philippinarum*. The consensus map which contained 9658 markers in 18 linkage groups was constructed through combing the male and female linkage maps.

A total of 435,272 markers were detected, of which 349,212 were polymorphic. After initial genotyping, a total of 21,689 markers that showed polymorphism in at least one parent and 95% of progeny were retained. Then the Mendelian fit test and genotyping percentage calculation were performed for further trimming. In total 10,873 markers that fit Mendelian ratios (*P* ≥ 0.001) and high genotyping rate (available genotype obtained over 115 progeny) were used to construct a sex specific map. Ultimately, 5348 and 5331 markers were used for female-specific and male-specific linkage map construction, respectively. The average read depth of genotyped markers ranged from 8.64 to 17.30 in the offspring. In the male and female parents, the average read depth was 36.96 and 30.01, respectively (Supplementary Table S4). Available markers were filtered for 95% integrity in each individual and chi-square tests with *P* < 0.001. Finally, 707 markers with hk × hk, 5233 markers with lm × ll, and 4933 markers with nn × np segregation types could be used for map construction. After data preparation, 5640 markers with types nn × np and hk × hk and 5940 markers with types lm × ll and hk × hk were used for the male and female map constructions, respectively. The group LOD value ranged from 4 to 14 depending on the linkage group. On the female map, 5348 markers fell into 20 linkage groups (LGs) and the genetic length was 1640.03 cM with an average marker interval of 0.62 cM (Supplementary Figure 1). On the male map, 5331 markers fell into 19 LGs; the genetic length was 1503.01 cM with an average marker interval distance of 0.62 cM (Supplementary Figure 2). Then the two parent maps were merged; the integrated map spanned 1926.98 cM with 9658 markers and fell into 18 LGs (Fig. 2). Among the 18 LGs, LG5 was the largest group, with a genetic distance of 137.8 cM and 546 markers. LG6 was the shortest group with 626 markers spanning 72.1 cM. The average marker interval ranged from 0.26 to 0.71 cM, with an average distance of 0.42 cM (Table 1). Between the markers, 4524 marker intervals were detected. Among them, 4500 marker intervals (99.5%) were less than 5 cM, 22 marker intervals were between 5 and 10 cM, and only two marker intervals were larger than 10 cM, which were on LG7 and LG8. In addition, we have conducted syntenic analysis between the map presented here and the clam genome through the BLAST search their sequence fragments against the clam genome (Supplementary Figure 3). The 9658 markers were used as anchors to orient these scaffolds. In total, about 1558 scaffolds were localized on the 18 LGs.

**Table 1:** Statistics of the linkage groups in constructed sex-averaged map of *R. philippinarum*.

Group	BIN markers	SNP markers	Map length (cM)	Average distance (cM)	Max. marker interval (cM)
lg1	288	575	110.37	0.38	4.86
lg2	289	689	100.5	0.35	4.42
lg3	277	624	119.02	0.43	5.54
lg4	346	733	105.57	0.31	5.26
lg5	266	546	137.77	0.52	6.16
lg6	280	626	72.07	0.26	4.46
lg7	346	752	132.67	0.38	19.02
lg8	267	572	114.86	0.43	19.62
lg9	333	714	101.19	0.3	5.8
lg10	268	580	117.77	0.44	7.21
lg11	132	351	72.48	0.55	4.45
lg12	221	434	89.38	0.4	3.59
lg13	204	461	118.58	0.58	5.94
lg14	278	571	109.32	0.39	6.39
lg15	154	320	105.33	0.68	7.32
lg16	247	526	120.74	0.49	6.14
lg17	199	385	95.4	0.48	4.64
lg18	146	199	103.96	0.71	9.18

#### QTL mapping of growth-related traits and shell color

**Figure 3:**
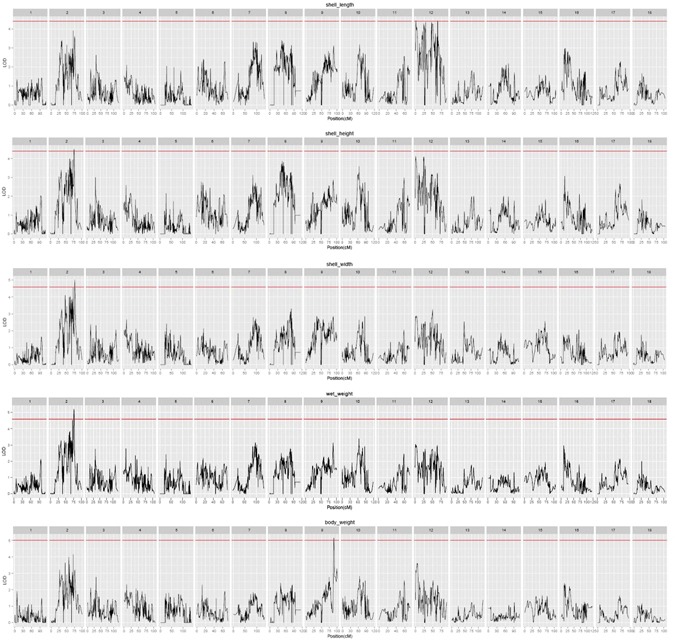
Growth-related QTL mapping and association analysis in *R. philippinarum* among all linkage groups.

**Figure 4:**
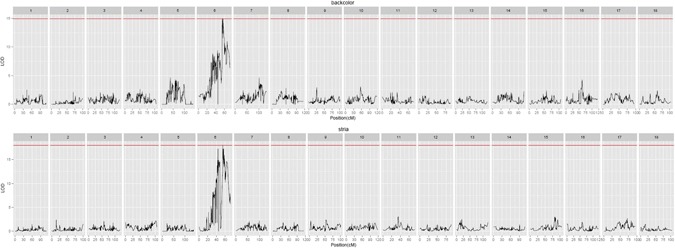
Shell color QTL mapping and association analysis in *R. philippinarum* among all linkage groups.

Multiple QTL model (MQM mapping) in the MapQTL package was used for analysis of the body weight information of 119 progeny. Body weight, shell length, shell width, and shell height followed a normal distribution. The estimated significant thresholds from permutation tests were 4.4 for shell length and shell height, 4.6 or shell width and wet weight, and 5 for body weight. Using the Composite Interval Mapping method, a total of 10 significant QTLs for growth trait and color were detected (Table 2). Three QTLs for shell length with the LOD score 4.46, 4.42 and 4.44, were located at 0.85, 63.31–63.88 cM of LG12 near marker lm2365, lm2492 and lm1421, and the proportion of phenotypic variation explained by these QTL was 4.16%, 4.16% and 4.17%, respectively. Thus, this region was considered to be a candidate genomic region involved in controlling the growth of *R. philippinarum* (Fig. 3). In addition, QTLs for shell height, shell width and wet weight with LOD score of 4.51, 4.66 and 5.21 were located at 75.7, 76.98 and 75.7 cM of LG2, respectively (Fig. 3). Interestingly, the QTLs for shell height and wet weight were located at the same position near the marker np1534. The other QTL for body weight was detected at 89.98 cM of LG9 near the locus np800 (Fig. 3). For shell color traits, the predominant QTLs for background color and stria were located at 55.14, 56.22, and 54.4 cM on LG6, with LOD scores of 14.97, 14.93 and 18.03, respectively (Fig. 4). The nearest markers for each QTL position are shown in Table 2.

**Table 2:** Detected QTLs for growth and color traits of *R. philippinarum*.

Trait	Group	Position	Locus	LOD	Variance	% Expl	Leftmarker	Rightmarker
shell_length	12	0.85	lm2365	4.46	4.16389	15.8	np1414	np233
shell_length	12	63.31	lm2492	4.42	4.17033	15.7	lm107	lm1926
shell_length	12	63.88	lm1421	4.44	4.16703	15.8	lm224	lm110
shell_height	2	75.7	np1534	4.51	2.46839	16	lm2065	np563
shell_width	2	76.98	np563	4.66	1.22276	16.5	np1534	lm834
wet_weight	2	75.7	np1534	5.21	0.140352	18.3	lm2065	np563
body_weight	9	89.98	np800	5.16	0.007729	18.1	lm404	lm2460
backcolor	6	55.14	np347	14.97	0.399897	44	np2178	lm2175
backcolor	6	56.22	hk442	14.93	0.400467	43.9	lm2174	lm200
stria	6	54.4	lm108	18.03	0.124216	50.2	lm918	lm1550

## Discussion

In this study, we constructed the first high-density genetic map of the marine clam aquaculture species *R. philippinarum*. Taking advantage of genotyping by sequencing technology, we were able to cost-effectively genotype 349,212 polymorphic markers in 121 clams (two parents and 119 offspring) from one full-sibling family. Such a high-density linkage map is expected to be a valuable resource for genomic analyses and fine-scale QTL mapping in the Manila clam *R. philippinarum*. GBS is a rapid, efficient, and cost-effective strategy for SNP development, genetic linkage map construction, marker-based complex trait selection, and draft genome assembly in many species with or without reference genomes^22,30–32^. Here, we used the combination of three restriction enzymes for GBS library construction. The combination of three restriction enzymes improved the efficiency of GBS by increasing the tag number, sequencing depth, and genome coverage, and also provided more chances to detect suitable regions for targeted fragments^20,22^. In the present work, we used a GBS approach to construct a high-density linkage map with 9658 markers spanning 18 linkage groups in the clam aquaculture species *R. philippinarum*.

Although *R. philippinarum* is one of the most important marine aquaculture species, little genetic information is available on this species, despite the fact that it has been cultivated in hatcheries since the mid 1970s^33^. In previous studies, the researchers focused mainly on the genetic characterization of populations and the evaluation of genetic differentiation and population structure^34–36^, followed by searches for molecular markers^37,38^, and evaluation of hybrids with congeneric species^39,40^. More recently, some studies related to the genetic response associated with the immune system, gene expression, and transcriptome have been reported^4–8^. However, the information is insufficient to generate a basis for developing genetic improvement programs. A high-density genetic linkage map can facilitate genome assembly and has been one of the fundamental components of genome sequencing^41^. Genome sequencing has been an important step for deciphering molecular mechanisms and accelerating genetic improvements of traits of interest in economically important species. High levels of polymorphism and heterozygosity have come to be considered universal hallmarks of most invertebrate genomes, particularly in marine mollusks^3,42^. In this study, 349,212 polymorphic markers were identified in the evaluated full-sib mapping family after GBS. The total number of polymorphic markers identified from *R. philippinarum* was relatively high compared to other mollusks^43^. The abundance of polymorphic markers implied the high number of polymorphisms, complexity, and heterozygosity features of the *R. philippinarum* genome, which will require special attention and consideration in the future whole genome assembly.

For the raw data, the read depths of all developed makers ranged from 8.64-fold to 36.96-fold. However, the data were filtered to exclude low read depth markers prior to mapping, so the average read depth of markers included in the linkage map was as high as 11.89-fold. The read depth of these markers ensured a high accuracy of marker genotyping. In this study, we adopted the pseudo-testcross strategy to construct the first high-density SNP genetic linkage map for *R. philippinarum* by taking advantage of parental heterozygosity^44^. The final consensus linkage map contained 9658 markers with a resolution of 0.42 cM (Table 1), comparable with other genetic linkage maps constructed through 2b-RAD (0.39–0.41 cM)^43,45^. In this study, the average intermarker distance was 0.42 cM in the sex-average map, which is much lower than that in previous linkage maps^43,46^. To our knowledge, this is the first high-density linkage map with the high density for *R. philippinarum* to date. Compared to the other linkage maps constructed using NGS technology for aquaculture species^47–49^, the intermarker distance was also shorter.

The manila clam is one of the most important species of commercial shellfish in aquaculture. Growth traits are of particular interest to *R. philippinarum* researchers due to their high production significance in the mollusk culture industry. QTL mapping represents an efficient approach for identifying the genetic loci underlying these traits, to allow marker-assisted selection to be applied in genetic breeding. QTL mapping of growth traits has been conducted in fish^50,51^, shrimp^52,53^, bivalve mollusks^43^ and many other aquaculture species. Such a high resolution map could be a powerful tool for QTL mapping. Three major QTL related to shell length was mapped to a 0.85 cM amd 63.31–63.88 cM region in LG12 in this study (Fig. 3). QTLs for shell height, shell width and wet weight were located at 75.7, 76.98 and 75.7 cM of LG2. The coverage and density of the current map are sufficient for us to effectively detect QTL for segregating traits, and two QTL positions were all coincident with the closest markers.

## Conclusions

In this study, a high-density genetic linkage map was constructed using the GBS method. The linkage map contained 18 linkage groups with a low intermarker distance. This high-density linkage map serves as a foundation of genetic knowledge for *R. philippinarum*. QTLs for growth related traits and shell color were identified and will be useful in marker-assisted selection studies for this important aquaculture species. These genomic resources may also play an important role in future whole genome sequencing projects and genetic breeding studies in *R. philippinarum*.

## Materials and Methods

### Mapping family preparation and DNA extraction

A total of three full-sib families were established in August 2014 through mating between a pair of common male and three different females. The parents were selected from wild population in Yingkou, Liaoning province. And the families were constructed in the Zhangzidao aquatic breeding farm in the city of Dalian, Liaoning province of China. One of these families exhibiting high within-family variation in growth traits was chosen for linkage and QTL analysis. A total of 119 one-year-old offspring were randomly selected from this family for linkage analysis and had the following traits recorded: shell length, shell width, shell height, body weight, wet weight, background color and stria. The mixed tissues from all specimens, including parents and offspring, were collected and stored in −80 °C for DNA extraction.

Genomic DNA of parents and progeny were extracted using a TIANGEN Marine animal DNA extraction kit (TIANGEN, Beijing, China). The concentration of extracted DNA was determined using a NanoDrop 2000 Spectrophotometer (NanoDrop, Wilmington, DE, USA). DNA integrity of each individual was evaluated by gel electrophoresis.

### GBS protocol and high-throughput sequencing

We used a GBS strategy in this study to develop SNP markers. First, we performed a GBS pre-design for restriction enzyme selection. In this step, the enzyme combination and sizes of digested fragments were predicted and evaluated. Generally, the following characteristics were required for the selection of appropriate restriction enzymes: (1) The digested tags should be evenly distributed throughout the sequences to be examined; (2) Repeated tags should be avoided; (3) The paired-end length of each tag should be suitable for the coverage of the Illumina HiSeq 4000 sequencing platform; (4) The tag numbers must be sufficient for the subsequent steps. To maintain sequence depth uniformity of different fragments, a tight length range of ∼50 base pairs (bp) was selected. Next, we constructed the GBS library according to the predesigned scheme. Here, three restriction enzymes were selected for DNA digestion and 0.1–1 μg of genomic DNA was incubated at 37 °C with *MseI* (New England Biolabs; NEB), T4 DNA ligase (NEB), ATP (NEB), and *MseI* Y adapter N containing barcodes, and then heat-inactivated at 65 °C. To further decrease the complexity and increase the sequencing depth and genome coverage, two additional enzymes, *NlaIII* and *EcoRI* (NEB), were simultaneously added into the *MseI* digestions to further digest the fragments at 37 °C. Then, the digested fragments with ligations were purified with Agencourt AMPure XP (Beckman) and subjected to PCR amplification using Phusion Master Mix (NEB) with universal primers as well as index primers to add index and complete i5 and i7 sequences. The PCR products were purified using Agencourt AMPure XP (Beckman) and pooled, then run on a 2% agarose gel. Fragments of 375–400 bp (with indexes and adaptors) were cut from the gel and purified with a gel extraction kit (QIAGEN). These purified products were then further cleaned with Agencourt AMPure XP (Beckman) prior to sequencing. Then, paired-end 150-bp sequencing was performed on the selected tags on the Illumina Hiseq X-Ten platform.

### Sequence data analysis

The sequence data from each individual were sorted according to the barcodes in the raw reads. To ensure that reads were reliable and without artificial bias (such as low quality paired reads, which resulted mainly from base-calling duplicates and adapter contamination) in the following analysis, raw data (raw reads) in FASTQ format were first processed through a series of quality control (QC) procedures with in-house C scripts. QC standards were as follows: (1) Reads with ≥10% unidentified nucleotides (Ns) were removed, (2) Reads with >50% of bases having a Phred quality <5 were removed, (3) Reads with >10 nt aligned to the adapter were removed, allowing ≤10% mismatches, and (4) Reads containing the *HaeIII* or *EcoRI* sequences were removed. Then, the Burrows-Wheeler Aligner (BWA) software^29^ was used to align the clean reads from each individual against the reference genome (settings: mem–t − 4 –k 32 –MR). Alignment files were converted to bam files using the SAMtools software^29^ (settings: –bS –t). If multiple read pairs had identical external coordinates, only the pair with the highest mapping quality was retained.

### SNP calling and genotyping

BWA was used to align the clean reads of each samples against the reference genome. If multiple read pairs have identical external coordinates, only retain the pair with the highest mapping quality. Then variants calling were performed for parents by using the GATK software: UnifiedGenotyper was used to estimate genotype and gene frequency. Unreliable snp were eliminated by filtering process. SNP calling was performed for parents and progeny using the SAMtools software^29^. The number of SNPs and types of transitions or transversions were counted. Then, a Perl script was used to filter the SNPs that had more than two genotypes. Polymorphic markers between the two parents were detected and classified into eight segregation patterns (ab × cd, ef × eg, hk × hk, lm × ll, nn × np, aa × bb, ab × cc, and cc × ab) according to the CP model in JoinMap 4.0 software^54^. For F1 population, hk × hk, lm × ll, nn × np were chose for genetic map.

### Linkage map construction and evaluation

Markers showing significantly distorted segregation (P < 0.001), integrity (<95%), or containing abnormal bases were filtered by JoinMap 4.0. The segregation patterns hk × hk and nn × np were used for the male parent map construction while the patterns lm × ll and hk × hk were used for the female parent map using JoinMap 4.0. The regression algorithm, three times circulation sequence, and Kosambi mapping function were used in marker distance calculation. The LOD value was set to 2.0–20. The integrated map for both the male and female parents was computed using the combine group for map integration function in MergeMap software^55^. A Perl script SVG was used to visualize exported maps, and heat maps were constructed to evaluate the maps.

### Data Availability

Data generated in this study is available in the Short Read Archive under NCBI BioProject accession number PRJNA450602.

## Electronic supplementary material

Supplementary Figures

Dataset 1

Dataset 2

Dataset 3

Dataset 4
