# Publisher Correction: Construction of a High-Density Genetic Map and Quantitative Trait Locus Mapping in the Manila clam *Ruditapes philippinarum*

**DOI:** 10.1038/s41598-018-28707-0

**Published:** 2018-08-07

**Authors:** Hongtao Nie, Xiwu Yan, Zhongming Huo, Liwen Jiang, Peng Chen, Hui Liu, Jianfeng Ding, Feng Yang

**Affiliations:** 1 Engineering and Technology Research Center of Shellfish Breeding in Liaoning Province, Dalian Ocean University, Dalian, 116023 China; 2 Key Laboratory of Mariculture and Stock Enhancement in North China’s Sea, Ministry of Agriculture, Dalian Ocean University, Dalian, 116023 China

Correction to: *Scientific Reports* 10.1038/s41598-017-00246-0, published online 22 March 2017

The original PDF version of this Article contained an error in the Published date ‘22 March 2017’ which was incorrectly given as ‘19 March 2017’.

In addition, a Data Availability statement has now been added to this Article.

## Data Availability

Data generated in this study is available in the Short Read Archive under NCBI BioProject accession number PRJNA450602.

These errors have now been corrected in the PDF and HTML versions of the Article.

