# Supplementary material for: Construction of a High-Density Genetic Map and Quantitative Trait Locus Mapping in the Manila clam Ruditapes philippinarum
Source: Sci Rep. 2017 Mar 22;7:229. doi: 10.1038/s41598-017-00246-0 (PMC5427961; doi:10.1038/s41598-017-00246-0)
Supplement: Supplementary file 1 — Supplementary Figures [file 41598_2017_246_MOESM1_ESM.pdf]

Construction of a High-Density Genetic Map and Quantitative Trait Locus Mapping in the Manila clam *Ruditapes philippinarum*

Hongtao Nie[1, 2], Xiwu Yan [1, 2] *, Zhongming Huo[1], Liwen Jiang[1], Peng Chen[1], Hui Liu[1], Jianfeng Ding [1], Feng Yang [1]

[1] Engineering and Technology Research Center of Shellfish Breeding in Liaoning Province, Dalian Ocean University, Dalian, 116023, China

[2] Key Laboratory of Mariculture and Stock Enhancement in North China's Sea, Ministry of Agriculture, Dalian Ocean University, Dalian, 116023, China

Correspondence and requests for materials should be addressed to X.Y. (email: yanxiwu@dlou.edu.cn)

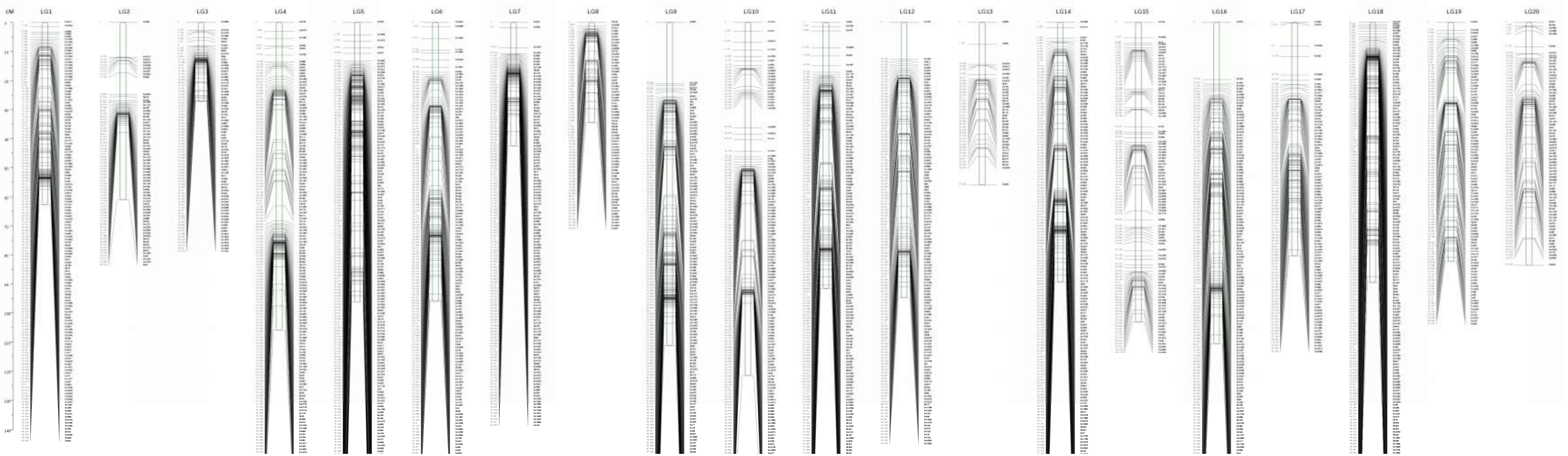

Figure S1 Female genetic linkage map of Manila clam *Ruditapes philippinarum*.

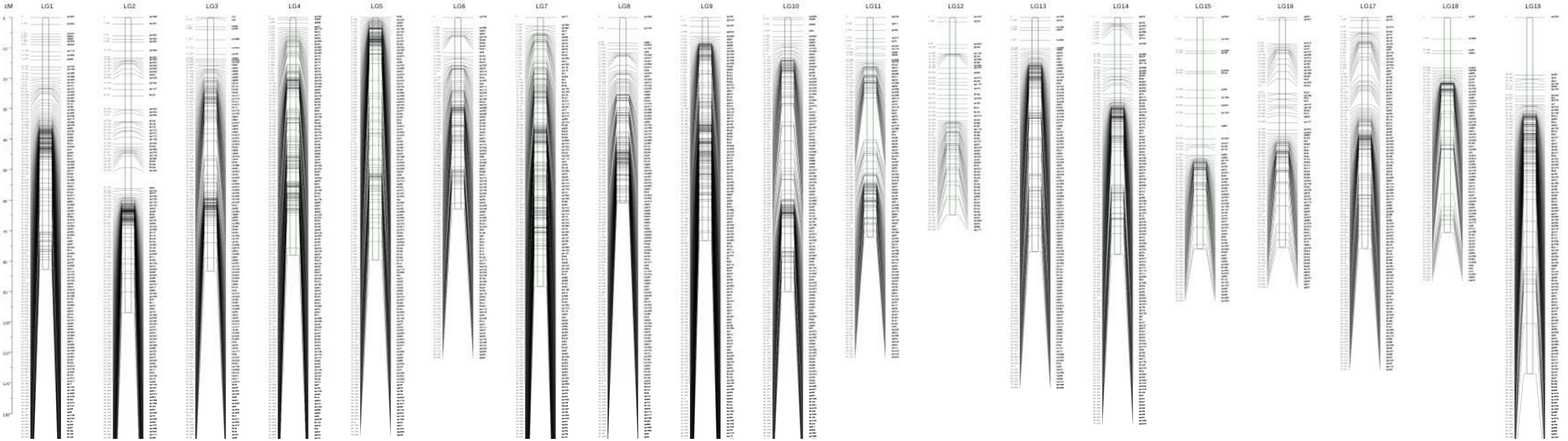

Figure S2 Male genetic linkage map of Manila clam *Ruditapes philippinarum*.

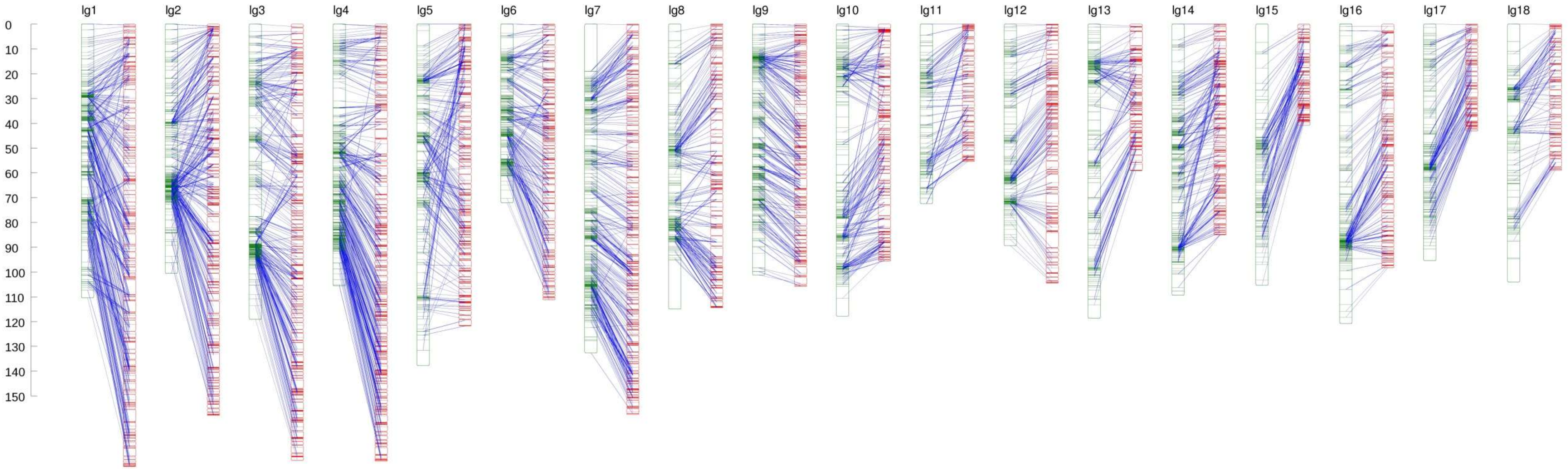

Figure S3 Syntenic analysis between the genetic linkage map and the clam genome.
